# Protective efficacy of the recombinantly expressed C-terminal domain of *Mannheimia haemolytica* leukotoxin in mice and goats

**DOI:** 10.2478/jvetres-2025-0056

**Published:** 2025-10-18

**Authors:** Thu-Dung Doan, Teerajet Laohasatian, Hsing-Chieh Wu, Chun-Yen Chu

**Affiliations:** International Degree Program in Animal Vaccine Technology, International College, National Pingtung University of Science and Technology, Pingtung, 91201 Taiwan; Innovative Bioproducts Technical Service Center, National Pingtung University of Science and Technology, Pingtung 91201, Taiwan; General Research Service Center, National Pingtung University of Science and Technology, Pingtung 91201, Taiwan

**Keywords:** bovine respiratory disease (BRD), cLktA, cross-strain efficacy, field-strain protection, murine challenge model

## Abstract

**Introduction:**

Bovine respiratory disease (BRD) complex is a leading cause of economic losses in the beef and dairy cattle industries. *Mannheimia haemolytica* is recognised as the primary pathogen associated with this disease. While antibiotics and vaccines are widely used against it, antimicrobial resistance and limited vaccine efficacy remain obstacles. *Mannheimia haemolytica* leukotoxin A (LktA) has been identified as a promising candidate for subunit vaccine development against BRD. However, the low expression and biological instability of the full-length LktA complicate its production. This study evaluated the immunogenic potential of the truncated LktA protein for subunit vaccine development.

**Material and Methods:**

Truncated proteins of LktA N-terminal (nLktA) and C-terminal (cLktA) were expressed in *E. coli* which were small enough for stable expression yet large enough to function as effective immunogens. The immunogenicity of the recombinant truncated LktA proteins was evaluated in mouse and goat models against a phosphate-buffered saline (PBS) negative-control group. Recombinant cLktA was emulsified with oil adjuvant and used to immunise mice and goats.

**Results:**

The cLktA group had significantly higher antibody levels at four weeks post-immunisation (wpi) than the PBS group. In goats, cLktA elicited high antibody responses up to six wpi. A single administration of cLktA conferred 80% and 100% survival against a *M. haemolytica* challenge.

**Conclusion:**

These findings show the C-terminal region of *Mannheimia haemolytica* LktA to be a highly immunogenic and protective antigen and suggest its potential as a candidate for subunit vaccine development.

## Introduction

*Mannheimia haemolytica* is a Gram-negative bacterium that primarily colonises the upper respiratory tract of healthy cattle, causing bovine respiratory disease (BRD) (23). This disease causes substantial economic losses in the cattle industry worldwide through reduced weight gain and increased treatment costs and mortality (1, 3, 6, 13). The disease costs up to USD 3 billion annually (19, 25, 30). Previous studies have estimated that approximately 50% of animals with lung abnormalities observed at slaughter show no clinical signs of BRD, highlighting the substantial proportion of subclinical BRD cases that may go unnoticed (35). The treatment of *M. haemolytica*-infected cattle with antibiotics is commonly practiced; however, this approach risks fostering multidrug resistance (31, 35). As a result, research efforts have increasingly focused on identifying and evaluating potential immunogenic candidates to create better vaccines. Efforts towards the development of new vaccines or the improvement of existing ones are crucial in combatting *M. haemolytica*. Notably, *M. haemolytica* leukotoxin A (LktA) has been identified as a promising antigen for subunit vaccine development against BRD (8, 7, 20).

Leukotoxin A belongs to the repeats-in-toxins family and represents the most critical virulence factor produced by *M. haemolytica* in the context of BRD (5, 12). This protein is initially synthesised as an inactive structural form (proLkt), which is subsequently activated by an enzyme encoded by the *LktC* gene. Proteins encoded by the *LktB* and *LktD* genes assist in secretion and stabilisation of the leukotoxin complex (15). Functionally, LktA is cytolytic, selectively targeting leukocytes such as macrophages and neutrophils, leading to impaired immune defences and severe tissue damage (26, 32). While LktA can bind to leukocytes from multiple species, including porcine, canine and equine hosts, it demonstrates the highest cytotoxicity to bovine leukocytes (33). Additionally, LktA binds to the leukocytes *via* lymphocyte function–associated antigen 1 (LFA-1), leading to Ca^2+^ influx into the cell, resulting in cell lysis (17). These features make LktA a promising antigen candidate for vaccine development, as its immunogenic properties can be harnessed to elicit protective immunity in cattle.

Structural and functional studies have identified that LktA protein contains two important parts: the N-terminus contains its toxic component and hydrophobic domain, and the C-terminus contains epitopes which are recognised by Lkt-neutralising antibodies (18, 24). At low toxin concentrations, *M. haemolytica* LktA stimulates cellular activation, while at higher concentrations, it leads to apoptosis-mediated cell death in bovine peripheral leukocytes and necrosis (27). Although the ability of LktA to activate the host immune system through its toxicity at low concentrations makes it an interesting candidate for vaccine development, its production faces significant challenges. The production yield of LktA is typically low during vaccine development (28), and full-length LktA of approximately 105 kDa in size is difficult to express in the *E. coli* system (15).

Therefore, our study aims to overcome expression difficulties by truncating LktA into N- and C-terminal regions designed to be small enough for stable expression yet large enough to function as effective immunogens. Both regions were evaluated to identify the immunogenic domain. The immunogenicity of the recombinant truncated LktA proteins was evaluated using both mouse and goat models.

## Material and Methods

### *Mannheimia haemolytica* cultivation and genomic DNA isolation

The Mh13948 strain from the Bioresource Collection and Research Center (Hsinchu, Taiwan) and the field-isolated B2 strain of *M. haemolytica* were cultured aerobically in brain heart infusion supplemented with 0.5% yeast extract and 3% horse serum at 37°C, being shaken at 150 rpm for 16 h. For isolation, 100 μL of bacteria suspension was spread on a new blood agar plate and incubated at 37°C for 24 h. Typical colonies from the culture-positive plates were then stained to check their reactions and cell shape under a microscope. A single colony of Mh 13948 was selected and cultured for further DNA extraction. Bacterial genomic DNA was extracted using the PetNAD Nucleic Acid Co-prep Kit (GeneReach Biotechnology, Taichung, Taiwan) according to the manufacturer’s instructions. The quality of the extracted DNA was confirmed by A260/A280 ratios between 1.8 and 2.0, and its concentration was determined using a NanoDrop spectrophotometer (Thermo Fisher Scientific, Waltham, MA, USA) to ensure adequacy for further manipulation. The extracted DNA was kept at −20°C for later use.

### Prediction of B cell epitopes of LktA protein

The sequence of the full-length *LktA* gene logged in GenBank under accession number AF314503 was retrieved from the National Center for Biotechnology Information. The potential linear B-cell epitope regions of the LktA protein were predicted using the Bepipred Linear Epitope Prediction 2.0 server (16).

### Plasmid construction and cloning of the N- and C-terminal proteins of LktA (nLktA and cLktA)

**Fig. 1.**
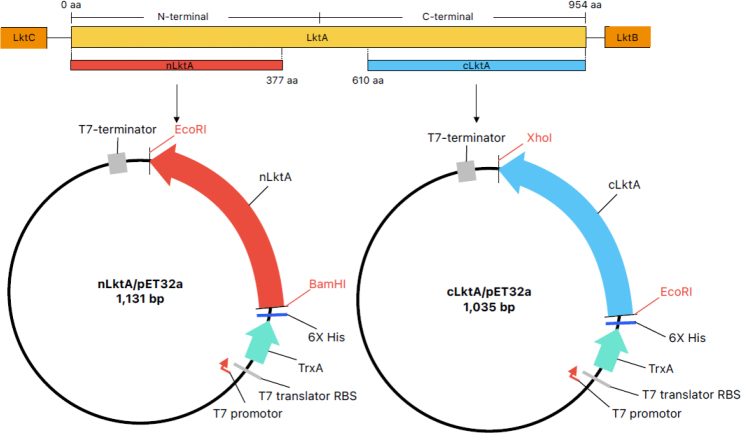
Constructions of pET32a/N- and C-terminal *Mannheimia haemolytica* leukotoxin A (nLktA and cLktA) plasmids. (A) Truncation of LktA. (B) Locations of the *BamHI* and *EcoRI* restriction sites for the N-terminal region of leukotoxin A (LktA) and of *EcoRI* and *XhoI* for the C-terminal region. LktB – leukotoxin B; LktC – leukotoxin C; aa – amino acids; bp – base pairs

Two recombinant protein constructs were designed in-frame with their vector using SnapGene version 3.2.1 software (GSL Biotech, Boston, MA, USA) (Fig. 1), and cloned into the pET32a plasmid vector (Novagen, part of Merck, Darmstadt, Germany) For cloning, the targeted genes were amplified using a PCR with the primers shown in Table 1. The expected PCR product for nLktA was 1,131 base pairs (bp) and for cLktA it was 1,035 bp. The PCR products were purified and then digested with *BamHI* and *EcoRI* for nLktA and with *EcoRI* and *XhoI* for cLktA. The digested genes were then purified and ligated into pET32a and selected in a lysogeny broth (LB) agar plate with ampicillin antibiotic. The final constructs were verified *via* restriction enzyme digestions, and sequencing was performed for reconfirmation.

**Table 1.** Primers for cloning *nLktA* and *cLktA*

Target gene	Sense	Sequence (5ʹ–3ʹ)	Restriction enzyme site	Length (base pairs)
*nLktA*	Forward	CGgaattcTCCCTTATGGGAACTAGACTT	*BamH*I	1,131
	Reverse	GCCctcgagTTATGCAGCAGCAGACACACC	*EcoR*I	
*cLktA*	Forward	AAAggatccAAACTTGGTGAAGGTGAT	*EcoR*I	1,035
	Reverse	AAActcgagTTAAGCTGCTCTAGCAAA	*Xho*I	

1 Lowercase portions of sequences are restriction enzyme sites

### Expression and confirmation of recombinant nLktA and cLktA proteins

Cells of *E. coli* BL21 (DE3) were transformed with pET32a/nLktA or pET32a/cLktA plasmids by heat shock (42°C for 45 s) for expression of nLktA and cLktA proteins. Transformed BL21 cells were cultured on LB agar plates supplemented with 100 μg/mL ampicillin, which served as a selective marker for the constructed plasmids. A single positive colony was propagated in LB medium with ampicillin until it reached an optical density (OD) at 600 nm of 0.4–0.6. Protein production at 0 and 4 h was collected after adding isopropyl ß-D-1-thiogalactopyranoside (Sigma-Aldrich, part of Merck, Darmstadt, Germany) at a final concentration of 1 μM/mL. After induction, the BL21 cells were collected by centrifugation at 5,000 rpm for 10 min. Empty pET32a was induced under the same conditions as the pET32a/nLktA and pET32a/cLktA constructs as a negative control. One mL of each induction of cell pellet was then mixed with sample buffer and heated at 100°C for 5 min for protein analysis *via* sodium dodecyl sulphate polyacrylamide gel electrophoresis (SDS-PAGE) and Western blot.

### Solubility screening and purification of cLktA

Before deciding whether to use a native or denaturing purification buffer, solubility screening was performed according to the instructions in the Bio-Scale Mini Profinity manual (Bio-Rad, Hercules, CA, USA). For soluble protein purification, induced cells were collected by centrifugation at 12,000 rpm for 10 min. Cell pellets from 100 mL of culture were lysed in native lysis buffer (300 mM KCl, 50 mM KH_2_PO_4_ and 5 mM imidazole) and then sonicated on ice for 10 min using a Vibra-Cell ultrasonic processor (Sonics & Materials, Newtown, CT, USA). The soluble protein was collected by centrifuging the lysate at 5,000 rpm for 10 min and then passing it through a 0.45-μM-pore filter. Subsequently, the filtered supernatant was purified with nickel-nitrilotriacetic acid beads according to the manufacturer’s protocol (Bio-Rad). The purified protein was confirmed by SDS-PAGE and Western blot. The purified cLktA was quantified *via* SDS-PAGE to a final concentration of 200 μg/mL using bovine serum albumin (BSA, KPL, Gaithersburg, MD, USA) as a standard. Serial dilutions of BSA were prepared to establish a standard curve. The proteins were run on a gel, stained with 0.1% Coomassie blue for 45 min and washed with destain buffer. The concentration of cLktA was measured using Image Lab 6.01 (Bio-Rad).

### Confirmation of recombinant proteins by Western blotting

To evaluate the antigenicity of the recombinant nLktA and cLktA proteins, sera from mice infected with an inactivated *M. haemolytica* B2 strain and secreted leukotoxin (sLkt) were used for binding with the recombinant proteins in a Western blot analysis. Briefly, the recombinant proteins were separated using 12% SDS–PAGE gel and subsequently transferred onto a polyvinylidene difluoride membrane (Merck, Darmstadt, Germany). The membrane was incubated with 5% skimmed milk at room temperature, shaken at 100 rpm for 1 h and incubated overnight at 4°C with anti-*M. haemolytica* B2 and anti-sLkt antibodies at final dilutions of 1 : 1,000. The membrane was washed with TBST 1× (PBS, containing 0.05% Tween 20) and incubated with goat anti-mouse horseradish peroxidase (HRP)-conjugated antibody (Arigo Biolaboratories, Hsinchu, Taiwan) at a dilution of 1 : 5,000 as the secondary antibody. The targeted protein was detected using Western Lightning chemiluminescent HRP substrate (Revvity, Groningen, the Netherlands) and analysed with a Gel Doc EZ Imager system run by Image Lab 5.0 software (Bio-Rad).

### Vaccine preparation and experimental animals

The purified cLktA was used to immunise the animals at a final concentration of 200 μg/mL. To formulate the inactivated Mh 13948 vaccine, *M. haemolytica* 13948 strain was cultured and then heated at 56°C for 30 min to reach a final concentration of 1×10^9^ CFU/mL. Phosphate-buffered saline (PBS) served as the negative control. All the antigen preparations were emulsified in Montanide ISA 201 VG water-in-oil-in-water adjuvant (Seppic, Puteaux, France) at a 1 : 1 (v/v) ratio. Vaccine sterility was assessed by inoculating 100 μL of each sample onto trypticase soy agar, thioglycolate agar and Sabouraud dextrose agar plates, which were incubated at 37℃ (first two media) or 25°C (Sabouraud) for 14 d. No viable contaminants were detected in any vaccine samples.

**Fig. 2.**
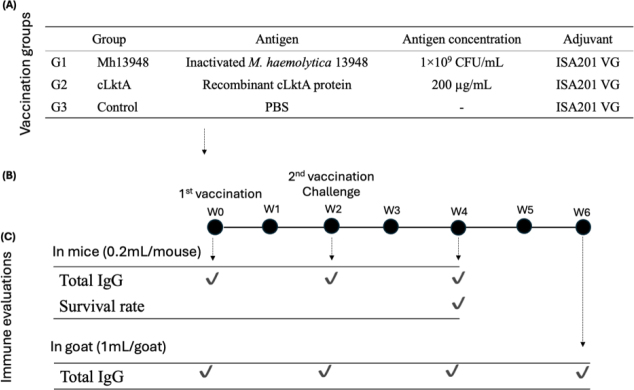
Antigens, immunisation schedule and immune evaluation of a truncated C-terminal leukotoxin A (cLktA) of *Mannheimia haemolytica* in mice and goat models. (A) Vaccination groups. (B) Immunisation schedule: primary vaccination administered in week (W) 0 and followed by a booster dose in W2. (C) Immune response evaluation through ELISA assay and challenge test. Mh 13948 – *Mannheimia haemolytica* strain 13948; CFU – colony-forming units; ISA201 VG – Seppic water-in-oil-in-water adjuvant 201; PBS – phosphate-buffered saline; IgG – immunoglobulin G

All experimental protocols were reviewed and approved by the Institutional Animal Care and Use Committee (IACUC) at the National Pingtung University of Science and Technology (NPUST). Mice were randomly assigned into three groups of 10 mice: group G1 were administered 1 × 10^9^ CFU/mL of Mh 13948), G2 received 200 μg/mL of cLktA and group G3 only PBS. Nine goats confirmed to be free of *M. haemolytica* antibodies were randomly assigned to three analogous groups G1, G2 and G3. Each animal received an intramuscular injection with a final volume of 0.2 mL for mice and 1 mL for goats. Two weeks after the primary vaccination, the animals received a booster injection. In mice, a challenge test was conducted *via* intraperitoneal injection of 0.2 mL of *M. haemolytica* (2 × 10^9^ CFU/mL) strain 13948 and the field-isolated B2 strain. The mice were monitored for clinical signs, and their survival rates were recorded over a two-week post-challenge period. The details of the vaccination schedule and immune evaluation are shown in Fig. 2.

### Detection of antibodies against *M. haemolytica*

To assess the antibody response induced by the vaccines, an indirect ELISA was conducted using sera from immunised animals at 0, 2, and 4 weeks post immunization and from goats also at 6 weeks. In brief, 96-well round-bottom plates were coated overnight at 4°C with 1.25 μg/mL of sonicated *M. haemolytica* 13948 in a coating buffer (15 mM Na_2_CO_3_, 35 mM NaHCO_3_ and 3 mM NaNO_3_, pH 9.6). The plates were then washed three times with TBST and blocked with 1% BSA for 1 h at 37°C under 80-rpm shaking. After blocking and washing, the diluted mice serum samples (1 : 300) and goat serum (1 : 800) were added to the plates and incubated for 1.5 h also at 37°C and under 80-rpm shaking. After the plates had been washed four times with TBST, HRP-conjugated rabbit anti-mouse IgG (Merck, Darmstadt, Germany) or rabbit anti-goat IgG (KPL) was added, and the plates were incubated at room temperature for 1 h. Next, the plates were washed five times with TBST before 100 μL per well of substrate solution (Peroxidase Kit; KPL) was added to allow colour development at room temperature for 10 min. Optical density was then measured at 450 nm using a Multiskan FC Microplate Photometer (Thermo Fisher Scientific, Vantaa, Finland), and the sample-to-positive (S/P) ratio was calculated as follows: S/P ratio = (OD _sample_ − OD _negative_) / (OD _positive_ − OD _negative_). An S/P ratio of 0.5 or greater was considered positive.

### Statistical analysis

Graphical data representations were generated using Microsoft Excel version 16.87, (Microsoft, Redmond, WA, USA) and statistical analyses were carried out with SPSS software version 22 (IBM, Armonk, NY, USA). Mean differences were evaluated using one-way ANOVA followed by Tukey’s *post-hoc* test. Results wer reported as the mean ± standard deviation (SD), with a significance threshold set at P-value < 0.05.

## Results

### Identification of B-cell epitopes of full-length LktA

**Fig. 3.**
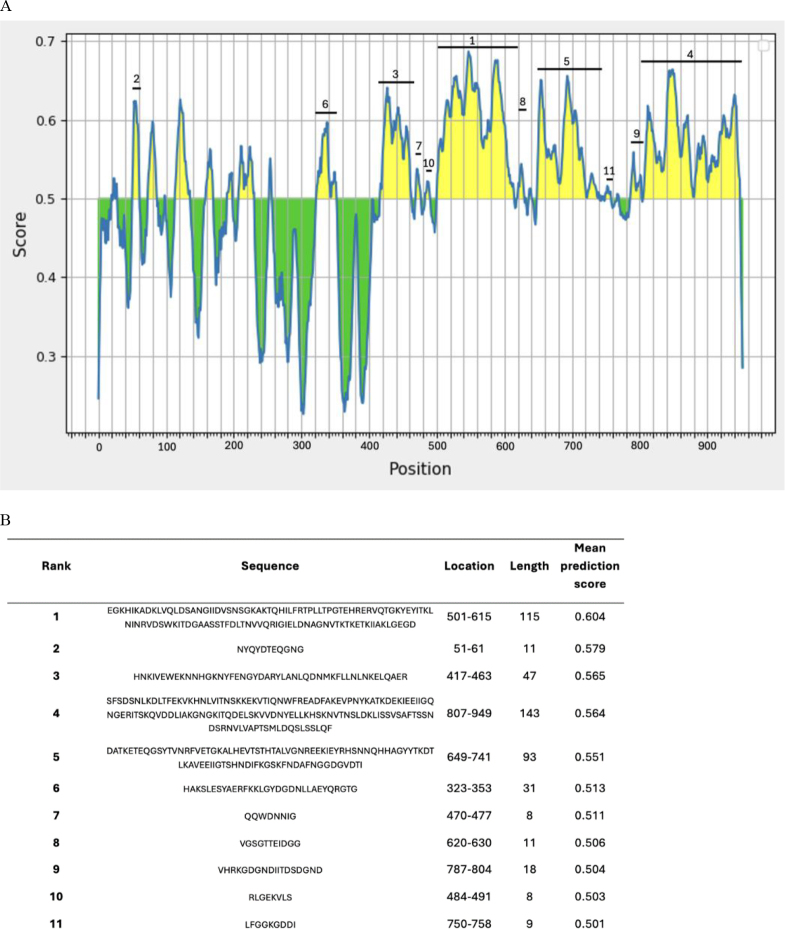
(A) Epitope prediction for the full-length LktA leukotoxin A protein of *Mannheimia haemolytica* with a threshold value of 0.5. Positive peaks above the threshold line indicate amino acids likely to be part of an epitope. (B) Eleven potential epitopes ranked based on their mean prediction scores, listed from highest to lowest (1–11)

The sequential linear B-cell epitope regions of the full-length LktA protein were identified (Fig. 3A).The LktA protein was found to predominantly show higher peaks in epitope prediction scores (0.5–0.7) in the C-terminus than in the N-terminus. This result indicated that the C-terminal region of LktA had a higher likelihood of being recognised by B-cells and appeared to be a key area for antibody binding (Fig. 3B).

### Confirmation of the nLktA and cLktA proteins

**Fig. 4.**
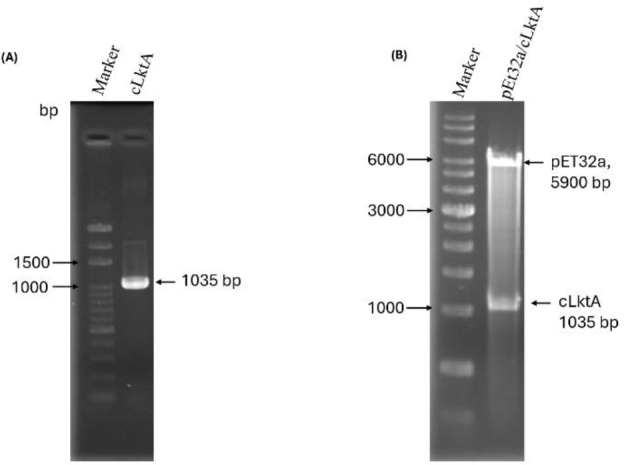
PCR amplification of C-terminal *Mannheimia haemolytica* leukotoxin A (cLktA) and confirmation of cloning cLktA into pET32a. (A) Product of 1,035 bp after amplification, indicating cLktA. (B) Restriction enzyme digestion with *EcoRI* and *XhoI* confirming the pET32a/cLktA construct

The n*LktA* and c*LktA* genes were successfully amplified using PCR, resulting in products of 1,131 bp and 1,035 bp, respectively (Fig. 4A). Restriction enzyme digestion for confirmation of successful protein construction revealed three fragments: pET32a (approximately 5,900 bp), nLktA (1,131 bp, data not shown) and cLktA (1,035 bp) (Fig. 4B).

### Analysis of recombinant nLktA and cLktA

**Fig. 5.**
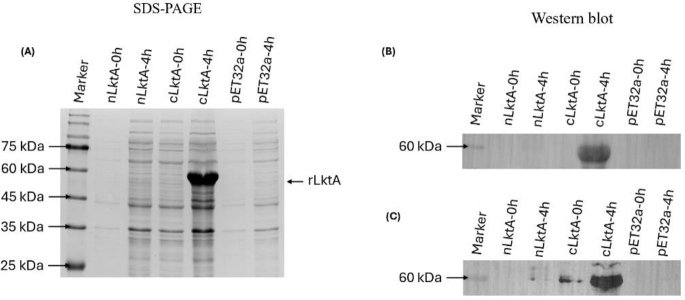
Expression of N- and C-terminal *Mannheimia haemolytica* leukotoxin A (nLktA and cLktA) in *E. coli*. (A) Isopropyl ß-D-1-thiogalactopyranoside-induced expression of nLktA, cLktA and control pET32a at 0 h and 4 h. (B) Western blot confirmation of recombinant cLktA protein using anti-*M. haemolytica* B2 strain antibody. (C) Further Western blot confirmation using anti–secreted leukotoxin (sLkt) serum

After protein expression, SDS-PAGE analysis showed prominent bands at the predicted molecular weight of 58 kDa for cLktA (pET32a inserts a 20-kDa Trx-His-S-enterokinase tag at the N-terminus). However, no protein expression was detected at the expected size of 61 kDa for nLktA (Fig. 5A). The recombinant protein’s identity was further confirmed by Western blotting using a 1 : 1,000 dilution of anti-*M. haemolytica* B2 (Fig. 5B) and anti-sLktA (Fig. 5C). The Western blot results demonstrated that cLktA exhibited strong antigenicity by effectively binding to sera from mice vaccinated with *M. haemolytica*. As a result, cLktA was chosen for further *in vivo* studies.

### Solubility screening of cLktA and vaccine preparation

**Fig. 6.**
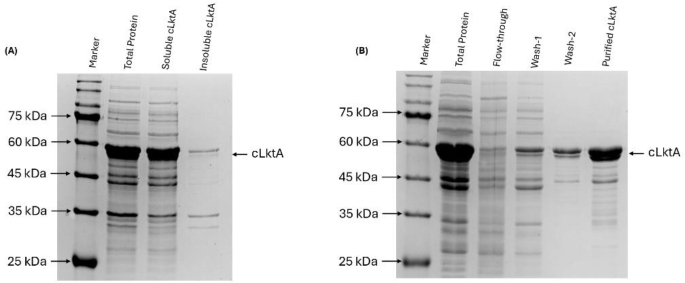
Purification of recombinant cLktA protein. (A) Solubility screening of cLktA demonstrating that it was a soluble protein. (B) Purification of cLktA using affinity chromatography

Regarding protein solubility, the cLktA protein was successfully expressed in a soluble form, as demonstrated in Fig. 6A. Following expression, the recombinant cLktA protein was purified as shown in Fig. 6B. The yield of cLktA was 700 mg/L.

### Humoral immune response to cLktA vaccine in mice and goats

**Fig. 7.**
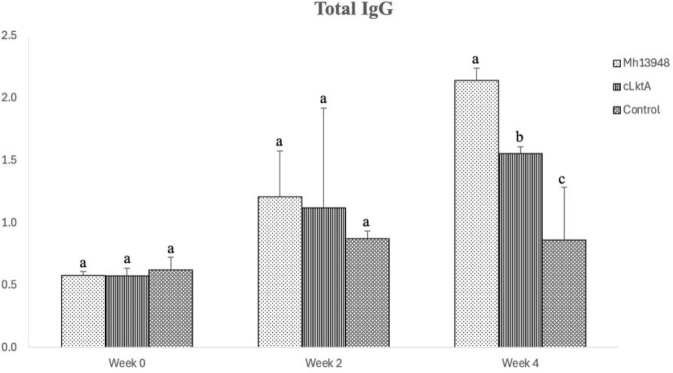
Results of indirect ELISA to determine the levels of antigen-specific total immunoglobulin G (IgG) in the sera of mice immunised with inactivated *Mannheimia haemolytica* 13948 (Mh 13948) or recombinant C-terminal protein of *M. haemolytica* leukotoxin A (cLktA) or injected with phosphate-buffered saline (control). Different superscript letters present statistically significant differences (P-value < 0.05)

For the mice experiment, at 0 and 2 weeks post vaccination (wpi), there was no significant different between the vaccinated and control groups. However, two weeks after the booster vaccination, both mice vaccinated with Mh13948 and those vaccinated with cLktA showed significantly higher antibody levels than the PBS group. While the cLktA vaccine group had significantly enhanced secretion of antibodies against *M. haemolytica*, this group’s production was not as high as that of the Mh 13948 vaccine group (Fig. 7).

**Fig. 8.**
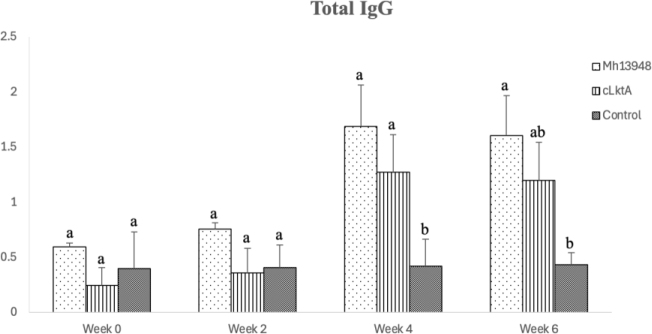
Results of indirect ELISA to determine the levels of antigen-specific total immunoglobulin G in the sera of goats immunised with inactivated *Mannheimia haemolytica* 13948 (Mh 13948) or recombinant C-terminal protein of *M. haemolytica* leukotoxin A (cLktA) or injected with phosphate-buffered saline (control). Different superscript letters present statistically significant differences (P-value < 0.05)

For the goat experiment, long-term antibody levels were measured up to 6 wpi. Similarly to the results observed in the mice experiment, no specific anti-*M. haemolytica* antibodies were detected in the immunised or control groups at weeks 0 and 2 wpi. However, goats vaccinated with cLktA produced significantly higher levels of specific anti-*M. haemolytica* antibodies compared to the control group from 4 to 6 wpi, indicating a sustained humoral response to the vaccines (Fig. 8).

### Survival rate of cLktA-vaccinated animals in the challenge tests

**Fig. 9.**
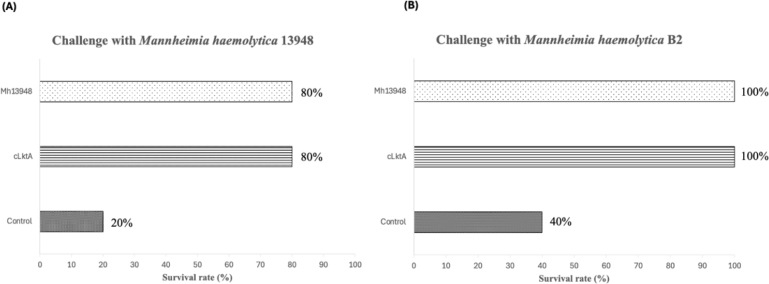
Survival rates of mice immunised with inactivated *Mannheimia haemolytica* 13948 (Mh 13948) or recombinant C-terminal protein of *M. haemolytica* leukotoxin A (cLktA) or injected with phosphate-buffered saline (control) when challenged with (A) *Mannheimia haemolytica* 13948 strain or (B) *M. haemolytica* B2 field isolate strain

Mice vaccinated with either the Mh13948 or cLktA vaccines demonstrated a survival rate of 80% following challenge with the homologous *M. haemolytica* 13948 strain, compared to a 20% survival rate in the PBS control group. Additionally, in a separate challenge with the heterologous *M. haemolytica* field-isolated B2 strain, vaccinated mice achieved a 100% survival rate, while the PBS group only had a 40% survival rate (Fig. 9). These results indicate that a single administration of cLktA vaccine provided protection against *M. haemolytica* challenges.

## Discussion

*Mannheimia haemolytica* leukotoxin A combined with bacterin is currently available commercially for protection against BRD. However, the full-length LktA protein is relatively large (approximately 105 kDa), making it challenging to achieve high yields in the *E. coli* expression system (28). The addition of a fusion protein from the expression plasmid further increases the molecular weight of the recombinant LktA, making it even larger than its naturally expressed form (9). In this study, the truncated C-terminal of LktA was successfully expressed in high yield as a soluble and functional protein. The antigenicity of cLktA was proved *in vitro* by Western blot, in which anti-*M. haemolytica* serum recognised the protein at its expected size of 58 kDa. Moreover, the mice and goats immunised with cLktA produced a strong and long-term specific antibody response against *M. haemolytica*. In the challenge test, a single administration of cLktA provided a high survival rate against the same *M. haemolytica* strain as the one from which the truncated protein came and perfect survival against a field-isolated B2 strain.

The N-terminus of *M. haemolytica* LktA may not be an immunogenic domain. Our *in-silico* result indicated that most of the immunogenic domains of LktA are located in the C terminus. Additionally, a previous report demonstrated that the deletion of the hydrophobic region in the N-terminal (amino acids 34–378) of LktA did not prevent it from binding to LFA-1, which is its receptor, but did prevent it from inducing biological effects (4). In an attempt to comprehensively evaluate the protective potential of LktA, both the N- and C-terminal regions were expressed for analysis. However, the expression level of nLktA observed in this study was low. This may be attributed to the high toxicity of the proteins in the N-terminal region of LktA. This toxicity may have negatively impacted host cell viability during overexpression, resulting in reduced viability and low protein yield (10, 22). Future codon optimisation should be considered to enhance the expression level.

The C-terminal domain of LktA contains a higher density of immunogenic epitopes. The C-terminal region was successfully expressed because of its highly hydrophilic residues. These residues improve its solubility and stability, helping it maintain its structure and function. Hydrophilic proteins are more likely to be exposed to and recognised by the immune system, facilitating the system’s easier targeting and memory formation. Our result demonstrated that cLktA elicited a high IgG titre and afforded better protection against *M. haemolytica*. This result is consistent with an *in-silico* epitope prediction analysis, which showed that most of the immunogenic epitopes of LktA were concentrated in the C-terminal region, particularly within the glycine-rich repeats and the area recognised by monoclonal antibodies (24, 29). We have made progress towards developing a subunit LktA vaccine against *M. haemolytica*, which causes BRD, and have proved that it can effectively stimulate a humoral response and confer protective immunity in mice; however, further evaluation is necessary. To prevent BRD, it will be important to assess the vaccine’s efficacy in the target host animal. Additionally, long-term antibody response and challenge tests should be conducted to comprehensively evaluate the candidate vaccine’s effectiveness.

With the advantage of its shortened recombinant protein, cLktA can be combined with other antigens to develop multivalent subunit vaccines. Studies have demonstrated that fusing the C-terminus of LktA with the PlpE protein from *Pasteurella multocida* significantly enhanced protection against bacterial challenge (2, 14). Furthermore, cLktA stimulates innate immunity by inducing inflammation and a robust cytokine response through the nuclear factor ĸ-light-chain-enhancer of activated B cells pathway (21, 37, 38). This property suggests its potential application as a protein-based adjuvant to improve vaccine efficacy (36).

## Conclusion

Overall, cLktA is a promising immunogenic antigen and protein-based biological adjuvant for vaccine development.
